# Microfluidic Biopsy Trapping Device for the Real-Time Monitoring of Tumor Microenvironment

**DOI:** 10.1371/journal.pone.0169797

**Published:** 2017-01-13

**Authors:** Angela Babetski Holton, Francy L. Sinatra, Jenny Kreahling, Amy J. Conway, David A. Landis, Soner Altiok

**Affiliations:** 1 Draper, Cambridge, Massachusetts, United States of America; 2 H. Lee Moffitt Cancer Center and Research Institute, Tampa, Florida, United States of America; 3 Department of Molecular Medicine, University of South Florida, Tampa, Florida, United States of America; Universita degli Studi di Catania, ITALY

## Abstract

The tumor microenvironment is composed of cellular and stromal components such as tumor cells, mesenchymal cells, immune cells, cancer associated fibroblasts and the supporting extracellular matrix. The tumor microenvironment provides crucial support for growth and progression of tumor cells and affects tumor response to therapeutic interventions. To better understand tumor biology and to develop effective cancer therapeutic agents it is important to develop preclinical platforms that can faithfully recapitulate the tumor microenvironment and the complex interaction between the tumor and its surrounding stromal elements. Drug studies performed in vitro with conventional two-dimensional cancer cell line models do not optimally represent clinical drug response as they lack true tumor heterogeneity and are often performed in static culture conditions lacking stromal tumor components that significantly influence the metabolic activity and proliferation of cells. Recent microfluidic approaches aim to overcome such obstacles with the use of cell lines derived in artificial three-dimensional supportive gels or micro-chambers. However, absence of a true tumor microenvironment and full interstitial flow, leads to less than optimal evaluation of tumor response to drug treatment. Here we report a continuous perfusion microfluidic device coupled with microscopy and image analysis for the assessment of drug effects on intact fresh tumor tissue. We have demonstrated that fine needle aspirate biopsies obtained from patient-derived xenograft models of adenocarcinoma of the lung can successfully be analyzed for their response to ex vivo drug treatment within this biopsy trapping microfluidic device, wherein a protein kinase C inhibitor, staurosporine, was used to assess tumor cell death as a proof of principle. This approach has the potential to study tumor tissue within its intact microenvironment to better understand tumor response to drug treatments and eventually to choose the most effective drug and drug combination for individual patients in a cost effective and timely manner.

## Introduction

The American Cancer Society reported in 2014, there was an estimated 1,665,540 new cancer cases diagnosed and 585,720 cancer deaths in the U.S. Cancer remains the second most common cause of death in the US, accounting for nearly 1 of every 4 deaths [1]. Current treatment options are based on large statistical sampling, which lack personalized therapeutic approaches for individual cancer patients. Hence, clinically relevant, reliable drug testing approaches would effectively match the patient to the best therapy in the shortest possible time. The complexity increases further as clinicians try to resolve these differences while prescribing treatment plans for each patient and their individualized disease. Thus, next generation treatments must clearly focus on patient specific responses to spare uncertain toxicity and additional non-effective prolonged courses of chemotherapy.

Cell-based assays and preclinical animal models have provided researchers platforms to discover both molecular and genetic biomarkers related to the progression of the disease. Elements of the tumor microenvironment have been shown to be instructive to tumor cells, making them more or less tumorigenic [2]. Genetic and epigenetic differences within each cell determine whether specific drug therapies will prove efficacious to each patient [3–5]. Tumor heterogeneity has proven culpable to the variability in response to chemotherapy. The spatial architecture and the resulting distribution of cells within the tumor can affect how they interact with each other and their microenvironment, impacting proliferation, differentiation, morphology and a range of cellular functions [6, 7]. Current in vitro chemosensitivity testing models, using cell lines or cloned cells selected from patient tumors, do not represent the tumor heterogeneity and the true microenvironment of the tumor. It has been demonstrated that the proliferation rate of the cells in a two-dimensional cell culture model are additionally 110–158% higher than that of three- dimensional cell culture format [8, 9]. Also, it is known that specific cancer drugs are more effective during the proliferative state [10]. Therefore, cancer cells grown in three-dimensional environments often show more resistance to cytotoxic agents than those grown in two-dimensional cultures [8]. More importantly, the cell culture conditions found in a conventional cell culture model may not be homogenous, primarily because of static and concentrated chemical gradients within the cell culture system. Chemosensitivity assays that use primary cultures of cells grown from surgically excised tumor specimens and incubated with a panel of therapeutic drugs are also performed in static conditions, often in a well-plate configuration [11, 12]. Limiting the tumor samples to a static environment hampers full nutritive support confounding the true efficacy of the drug treatment. Further, most biopsy driven models offer only an end-point analysis of how the cancer cells respond to treatment without regard to each samples own baseline health prior to the initiation of the drug exposure [13]. The resulting poorly defined extracellular conditions in a cell culture system can hamper precise quantification of the link between cellular responses and the tested conditions [14]. Although chemosensitivity testing is an intuitively attractive concept, to date they have failed to demonstrate clinical efficacy to predict tumor responses in vivo, mainly because the ex vivo growth of tumor cells in static well plate conditions may lead to artificial selection of specific tumor subpopulations depending on culture conditions, thereby limiting ultimate clinical correlation [15, 16].

Tumor is a heterogeneous tissue receiving continued stimuli from its microenvironment where the cancer cells dynamically shift their response to both chemical communications and mechanical forces in time and space. Therefore to accurately assess a tumor’s response to drug therapy, the microenvironment’s response requires a dynamic but controlled and measured approach for multiple cell types over a variety of time points and conditions [17, 18]. Through the use of microfluidics, cultured environments can be controlled to homogeneous microenvironments with low chemical gradients [19–21]. Unlike a static cell culture, a perfusion cell culture model can provide a system for continuous nutrient supply and removal of metabolic products, thereby maintaining a more stable culture environment [14, 22]. Additionally, a controlled environment offers the additional advantage of being capable of creating hydrodynamic forces, such as shear stress, in the physiological range as seen in vascular capillaries and tissues known to have Reynolds numbers <100, resulting in predominantly diffusion based characteristics, over spatial distances of approximately 100 μm [23, 24] within which cellular metabolite uptake, gaseous exchange and waste removal occurs [25]. Microfluidics are low cost, micro-sized footprints that offer high sensitivity with high throughput results while having the advantage of temporal control of physics, biology, chemistry and physiology at the cellular level [26]. Small dimensions, low burden on reagent use with controllable and well-defined culture conditions make microfluidics valuable for drug testing studies.

Recent in vitro microfluidic approaches have attempted to recapitulate some of the tumor architecture but relied mainly on cancer cell lines that lack important phenotypes of the primary cancer cells due to prolonged culture in serum with growth and attachment on tissue culture plastic; not representative of the parent tumor. Three-dimensional gels derived from animals such as bovine collagen are variable in composition [27, 28] and can pose variability between samples grown in their presence. Synthetic hydrogels often require post processing with radiation, ultraviolet, or chemical catalysts which may be toxic to the cells [28, 29]. Additionally, artificial three-dimensional scaffolds, contain less of the essential stromal elements such as extracellular matrix (ECM) component and stromal cells found in the tumor microenvironment in vivo and therefore do not support an environment for native cellular behavior. The interaction between cells and the ECM is intimate and dynamic. The ECM applies mechanical forces that guide cellular organization [30] and directs cellular behavior and differentiation through inherent signaling proteins and or its diffusion properties of external and intercellular signaling [31]. Cells also can respond to their local environment, remodeling local ECM, by degrading or synthesizing new ECM elements [32]. Additionally, the diffusion of nutrients, metabolic products and soluble cytokines is critically controlled in tumors and difficult to mimic using artificial three-dimensional matrix materials. For example there is no distinct microporous structure in hydrogels thus, mass transport mainly depends on slow diffusion through submicron-sized pores. The density of the gels can cause variations in mass transport of oxygen and nutrients throughout the sample [33] and may influence the hydrodynamic properties of the microfluidic channel creating increased resistance to flow by occluding a greater percentage of the channel. Variations in the microenvironment of the cellular samples and fluidic properties of the device further complicate analysis of drug effects on platform. Still, the high water content (95.0–99.5%) enables sufficient exchange of essential biomolecules and metabolic end-products to maintain cell viability in a limited thickness but at the same time offers weak mechanical properties [34]. Static or diffusion-based fluidic models are provided by reservoirs on or off chip or with the fabrication of microchambers or cavities for the cell structures and nutritive media supply [35]. Other microfluidics systems deliver pulsatile flow with periods of flow and no flow or pulsations intermittently for purposes of nutritional and or drug delivery therefore delivering pressures and shear at varying levels [13, 36]. Fluid shear stress has important consequences on the proliferation dynamics of cancer cells [37–39]. Applied shear resulting in the deformation of the cell membrane, leading to changes in signaling cascades, affects not only the cytoskeleton but also gene transcription that ultimately affects cell growth, survival and response to therapeutic agents.

One of the main challenges in the development of in vitro assays to predict tumor response to treatment is the availability of fresh tumor tissue obtained from patients. We have previously described a novel short-term ex vivo assay to explore pharmacodynamic predictors and indicators of response to anticancer drugs by using tumor fine needle aspiration biopsy (FNAB) samples. The patient-derived pre-clinical xenograft mouse studies and the results of the clinical studies performed by our group demonstrated that the short term ex vivo assay has a high negative predictive value and provides pharmacodynamic data to identify patients whose tumor would likely respond to treatment [40]. To add to this ex vivo approach we have developed a novel three-dimensional microfluidics approach using patient tumor samples obtained by FNAB that provide micron-scale tumor tissue where the interaction between tumor and its microenvironment are maintained in their biological configuration. The device was fabricated from polydimethylsiloxane (PDMS) by a photolithography process having 10 channels each with a centralized array of oblong posts that trap the biopsy when delivered into the device by injection. The biopsy sample is held in place while the device is primed with fresh media. The device is high- throughput in which each channel may be fed by a separate reservoir or multiple reservoirs allowing single agent or combinatorial treatment regimens that have the capability to be also temporally altered throughout the progression of treatment. FNAB tumor samples are added to the system and analyzed for their baseline health prior to the onset of treatment. The FNAB tumor samples are then monitored throughout the treatment period for progression of death through apoptosis and viability signified by detection of esterase activity within the intact cell.

We hypothesize that a re-circulating multi-trap multi-channel microfluidic device capable of introducing multiple drug combinations to patient derived biopsies will maintain the microenvironment of the tumor and may recapitulate in vivo tumor response to drug treatment, which ultimately can be used for personalized cancer therapy. Herein, we demonstrate a cytotoxic drug model using a known protein kinase C inhibitor, staurosporine [41], to optimize the microfluidic device towards a potential clinically relevant chemosensitivity assay.

## Materials and Methods

### Microfluidic device design and fabrication

**Fig 1:**
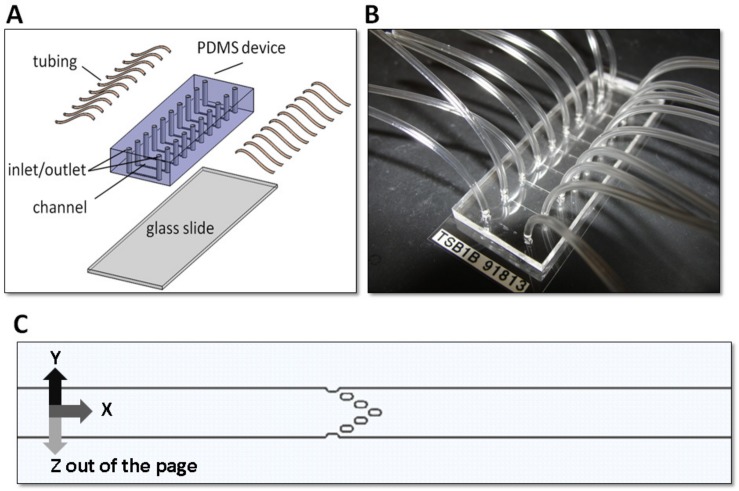
Microfluidic device design. (A) Exploded view of the device showing the main body made from PDMS containing the 1 mm diameter inlet and outlets punched for each of the 10 channels. The main body is then sealed to a 25 x 75 mm glass slide by plasma gas treatment. Tubing is inserted into each of the inlet and outlets of the device. (B) Fully assembled PDMS prototype. (C) Solid Works rendering of a channel in the device showing the central post arrangement used to trap each FNAB tissue sample. The channel is 10 mm long, 600 μm wide and 125 μm in height. Each post is 150 μm long, 75 μm wide and 125 μm in height.

The design of the microfluidic network was created in L-Edit with the dimensions of the 10 microfluidic channels, trapping posts and inlets and outlets. A transparency mask was created from the design file printed by a high-resolution printer. The transparency mask was used in a soft-photolithography process using tridecafluoro-1,1,2,2-tetrahydrooctyl trichlorosilane (Gelest Inc, Morrisville, PA), photoresist to create a silicon wafer master. The silicon wafer was patterned with tridecafluoro-1,1,2,2-tetrahydrooctyl trichlorosilane using standard photolithography techniques to create a mold for forming the channels and traps. Uncured PDMS (10:1 base to curing agent weight ratio) was cast on top of the wafer to a thickness of 6 mm followed by degassing and curing at 65°C for 12 hours. The cast PDMS was peeled from the mold and fluidic inlet/outlet ports were made using a 1 mm biopsy punch for each channel. Devices were cleaned with 70% isopropanol, rinsed with sterile filtered H_2_0 and further rid of debris by filtered forced air. The PDMS surface of the device was rendered hydrophilic by exposure to oxygen plasma (Plasma Etch PE-100 Asher, Carson City, NV at 100 mTorr, 10 s, 150 W) to facilitate scaffold loading of a glass slide that seals the channel of the device. Finally the device was sterilized by ethylene oxide gas and kept in their sterilized pouches until use. Fig 1 demonstrates the overall device features and the trap configuration in the 10 channels of the device.

### Establishment of patient-derived xenograft

This study was approved by the University of South Florida’s Institutional Animal Care and Use Committee (IACUC) and Institutional Review Board. All animals were maintained and evaluated in accordance with IACUC standards care in pathogen-free rooms at the H. Lee Moffitt Cancer Center (Tampa, FL). Patient derived xenograft models are established using fresh tissue obtained at the time of resection from patients with lung, melanoma and bladder cancers and established as an F1 generation through subcutaneous implantation into four-week-old female immunodeficient hairless Crl:SHO-Prkdc-SCID-Hr-hr mice (Charles River Laboratories). The patients provided written informed consent for the study. The tumors from this initial passage are harvested and implanted subcutaneously into F2 generation. Tumors are allowed to grow to a size of 1.5 cm, at which point they are harvested, divided into small 3 × 3 × 3 mm pieces, and transplanted to an F3 generation. At the end of the experiment protocol, the animals were sacrificed by cervical dislocation after carbon dioxide sedation.

### FNAB procedure and loading FNAB samples on device

FNAB was performed using a 25-gauge needle where the subcutaneous tumor was sampled from all areas of the tumor, as previously described [42, 43]. Following the procedure, a Diff-Quik (DQ) stain was performed on the smear samples to both validate viable tissue was captured in the biopsy and also characterization of the pathology of the cancer. FNAB tumor samples were received immediately after biopsy procedure and transported on ice in RPMI media (RPMI 1640, phenol red and sodium bicarbonate powder 25 mM HEPES, 10% heat inactivated fetal bovine serum, 1% (w/v) penicillin/streptomycin, 0.3 g/mL L-glutamine, sterile filtered before use). The FNAB tumor sample was centrifuged at 200xg for 10 minutes discarding the supernatant and resuspended with fresh media. FNAB tumor fragments measuring ~300 μm in diameter were selected for introduction into the device. FNAB fragments are elastic and therefore able to deform to the confined space of the channel dimensions. FNAB tumor samples were placed in the reservoirs attached to the peristaltic pump and perfused with complete media at a flow rate of 125 μL/hr. Media and or drug were retained in reservoirs individual to each channel covered with a 2 μm pore gas permeable membrane open to atmospheric conditions allowing oxygen to dissolve into media. The percent of dissolved oxygen in media was validated in prior multi-day experiments on platform with oxygen sensors (NEOFOX-KIT-PROBE, Ocean Optics). Additionally, the amount of oxygen that could be supplied into a PDMS device per day, based on our device dimensions, was estimated to be 3.5x10^-5^mol O_2_/device day [44, 45]. The oxygen within PDMS per day is sufficient to sustain FNAB biopsy with roughly 1000 cells with a total respiration of sample to be 8.64x10^-9^mol/day. Therefore, adequate oxygen is delivered to the FNAB sample per day if the system was stagnant but because the media is additionally renewed with flow rates of 125 μL/hr, oxygen is renewed more often in the device. The temperature of the system was maintained at 37°C by a microscope incubation chamber.

### Evaluation of small molecule drug perfusion in FNAB

Doxorubicin HCL purchased from Sigma Aldrich was reconstituted with 10% dimethyl sulfoxide (DMSO) to a concentration of 1 mg/mL. A working concentration of 1 μM, diluted with cell culture media, (RPMI 1640, without glutamine, phenol red and sodium bicarbonate powder) was made by adding 25 mM HEPES, 10% heat inactivated fetal bovine serum, 1% (w/v) penicillin/streptomycin, 0.3 g/mL L-glutamine sterile filtered before use. The 1 μM doxorubicin solution was delivered by peristaltic pump at a flow rate of 125 μL/hr for a 24-hour period. Fluorescent imaging was performed by a Zeiss Axio ObserverZ.1/ApoTome.2 inverted scope over a 24-hour period to measure drug perfusion throughout the FNAB sample in device.

### Evaluation of large molecule perfusion in FNAB

A perCP/Cy5.5 conjugated EpCAM mouse anti-human monoclonal antibody (VU-1D9, abcam) and a FITC conjugated CD44 mouse anti-human (clone G44-26, BD pharmingen^TM^) and their respective isotype controls, mouse IgG1 monoclonal MOPC-21 (perCP/Cy5.5, abcam) and Dnk pAb to Ms IgG (Dylight®488, abcam) were diluted with RPMI media to a working solution 1:200. FNAB tumor samples were loaded into nine separate channels. The antibody solution containing both the EpCAM and CD44 was perfused into three channels. The isotype control antibodies, used to evaluate for nonspecific binding, were perfused into three other channels. The last three channels contained media only to serve as background auto-fluorescence controls for tissue. One channel was not needed and therefore not used for this experiment. A flow rate of 125 μL/hr was used for all channels. Fluorescent imaging was performed by a Zeiss Axio ObserverZ.1/ApoTome.2 inverted scope using a 378HE Green Fluorescent Protein (GFP) filter (excitation: 450-490nm /emission: 500-550nm) and a 45 Texas Red (TR) filter (emission: 540-580/excitation: 593-668nm) over a 24-hour period.

### Staurosporine treatment

Staurosporine was reconstituted with DMSO to a stock concentration of 10 mM. A further working solution of 50 μM was made diluted with RPMI media. Two experimental groups were tested, a negative vehicle control group; media only with 0.1% DMSO, and a 50 μM staurosporine treated group. FNAB tumor samples were tested in triplicate for each group across 6 test sessions. A total volume of 1.5 mL in each reservoir containing either test condition was discretely circulated through each channel of the device containing a single FNAB tumor ~300 μm fragment for a treatment period of 5 days.

### Measurement of viability and death

Calcein AM viability stains (Molecular Probes) and CF594-Annexin V (Biotium) antibody were both added to cell culture media with binding buffer for final concentrations of 5 μM for each dye. The dye solution was then used to perfuse FNAB tumor samples to assess for viability and apoptosis in real time continuously over the time course of experiments. Fluorescent imaging was performed by a Zeiss Axio ObserverZ.1/ApoTome.2 inverted scope using a 378HE Green Fluorescent Protein (GFP) filter (excitation: 450-490nm /emission: 500-550nm) and a 45 Texas Red (TR) filter (emission: 540-580/excitation: 593-668nm) for Calcein AM and CF594-AnnexinV respectively. ZEN 2012 software was used to calculate the sum of intensity units throughout the FNAB tumor fragments in each device channel. Background and or auto-fluorescence were subtracted out from each summed value of intensity. The sum of intensity values for both Calcein AM and CF594-AnnexinV were normalized for each FNAB tumor fragment by dividing the summed values of intensity of each emission by the size (area μm^2^) of the FNAB tumor fragment in each z-axis throughout the volume of the tumor fragment to give total relative fluorescent units (*tRFUs*) for each FNAB tumor fragment. The health of each FNAB tumor fragment at the end of each drug trial was reported as a viability index (*Vi*) = GFP *tRFUs* (viability) / TR *tRFUs* (apoptosis) to normalize all samples across all test sessions.

### Statistical analysis

The mean and standard error of each treatment group was calculated with n = 18 samples. A repeated measures ANOVA analysis was performed for Within-Subjects Effects across the 5-day testing period for both groups. A Greenhouse-Geisser correction was performed for violation against sphericity. Sphericity is an assumption that the variances of the differences between all possible pairs of groups are equal. If sphericity is violated, then the variance calculations could result in an inflated F-ratio with the proportion of rejections of the null hypothesis larger than the α-level when the null hypothesis is true. In order to minimize this problem, Greenhouse-Geisser procedure corrects the deviation to sphericity by using an index to correct the number of degrees of freedom of the F-distribution [46]. To determine the significance between groups, a Student’s two-tailed t test was performed from the calculated mean and standard deviation of each treatment group. P-values less than 0.05 were considered statistically significant.

## Results

### Evaluation of small molecule drug perfusion through the FNAB on device

**Fig 2:**
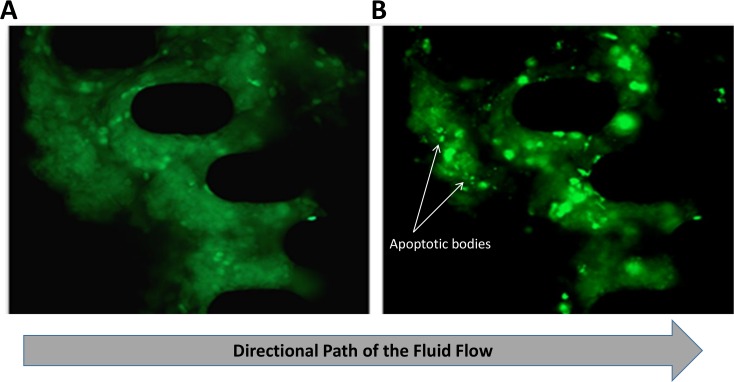
Assessment of small molecule drug perfusion in a tumor FNA fragment using Doxorubicin HCL. (A) Drug perfusion after an 8-hour period showing distribution of drug indicated by green fluorescent intensity spatially similar throughout the FNAB tissue sample. (B) Drug perfusion after a 24-hour period showing intensity peaks spatially similar throughout the FNAB tissue sample and the formation of apoptotic bodies.

A model of fluid flowing in the channel of the microfluidic device was designed to understand how various parameters such as flow rate, channel dimensions and tumor size would affect the FNAB tissue sample in the designed trap (S1 Text). To assess the shear stress on a tumor, we simulated a tumor, trapped inside a channel by the posts (S1 Fig). To assess the accuracy of the simulation model performed with multiphysics software licensed from COMSOL, we tested the fluid profile through the device with 1μm diameter polystyrene beads conjugated to a fluorophore with an excitation/emission wavelength of 580/605 nm (S1 Text). With concerns of lack of interstitial flow at the tumor interface in the center of the channel, seen from results of the fluorescent bead assay with FNAB tumor fragment in channel (S2 Fig), we next tested the advective properties of fluid flow through FNAB tumor samples using a homogenous fluorescent solution. We predicted that the size of the 1μm diameter fluorescent beads, previously used, did not accurately depict homogenous fluid flow. Therefore, we opted to use a soluble small molecular weight compound to track interstitial flow and drug penetration throughout the FNAB tumor fragment. We selected Doxorubicin HCL (Sigma Aldrich, USA) for its natural fluorescence in a spectrum similar to FITC with an excitation of 480 nm and an emission of 560–590 nm [47, 48]. We hypothesized, if the bead assay was correct and the fluid path was restricted by the FNAB tumor fragment in channel with lower to no flow velocities in the center of the channel and higher velocities at the side walls of the channel, there would be evidence of fluorescent gradients of Doxorubicin seen throughout the FNAB tumor fragment. Fluorescent intensities would be weak or nonexistent with little to no drug in cells of the tumor fragment residing in the central portion of the channel and higher intensities with greater drug concentrations in cells of the tumor fragment residing nearest the sidewalls of the channels. Secondly, with flow restriction, the infusion of drug into cells would occur first in areas of the tumor that experience greatest flow, specifically, at the tumor interface where the tumor fragment first meets the fluid front. The cells located in the backside of the tumor fragment, specifically at the center of the channel, would experience a delay in drug infusion due to the lag time in flow and low to no flow velocities as demonstrated by the fluorescent bead assay. To test for full tumor drug penetration we selected lung adenocarcinoma FNAB tumor samples taken from patient derived xenograft mice as previously explained. Doxorubicin was introduced into a reservoir of cultured media at a working dilution of 1μM. The drug solution was pumped into the channels with FNAB tumor fragments in trap for a period of 24 hours. Over the 24-hour period, continuous fluorescent imaging results indicated a gradual uptake of the drug into the cells demonstrated by an influx of green fluorescence into distinct clearly defined semi-circular membrane bound cells. Fluorescent intensities of the drug were evenly distributed across the tumor sample occurring simultaneously in all areas of the tumor throughout the 24-hour period (Fig 2A). Excitingly, we were also able to capture resulting apoptotic cellular behavior during the 24-hour period of drug infusion. Apoptosis was signified by chromatin clumping and condensation with nuclear fragmentation and the formation of apoptotic bodies evident from high intensity concentrations of the dye and foreign body formation [49] (Fig 2B). Apoptosis was seen in all regions of the tumor sample and occurred simultaneously at opposing ends of the flow both in cells located in the center of the tumor sample and at the sidewalls.

### Evaluation of large molecule protein antibody perfusion through the FNAB on device

**Fig 3:**
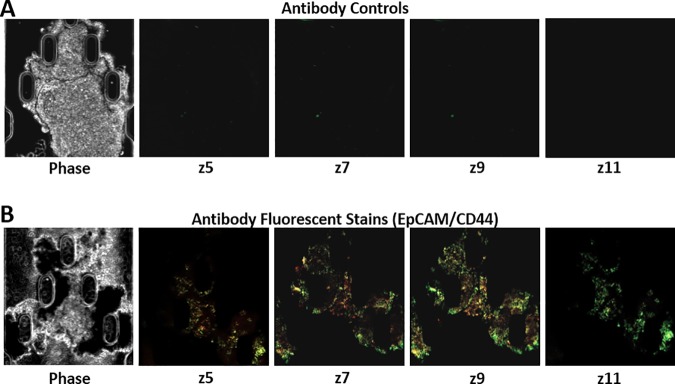
Evaluation of antibody perfusion through the tumor FNAB on device. (A) 10x Phase contrast image of FNAB sample in trap of device and 10x fluorescent z-axis images (z5, z7, z9, z11) 24 hours post the staining procedure using isotype control antibodies for both EpCAM (red-Cy5) and CD44 (green-FITC). (B) 10x Phase contrast image of FNAB sample in trap of device and 10x fluorescent z-axis images (z5, z7, z9, z11) 24 hours post the staining procedure using EpCAM (red-Cy5) and CD44 (green-FITC).

Full drug perfusion through the full FNAB tumor fragment was successfully demonstrated with a small molecular weight compound solution of doxorubicin HCl. The next step was to determine if larger molecular weight protein based molecules could penetrate the FNAB tumor sample during fluid perfusion. To demonstrate large molecule profusion through FNAB tumor samples we used protein antibodies that warranted specific recognition of a protein/protein interaction with the tumor tissue. An antibody, conjugated to Cy5 fluorophores, raised against EpCAM that is expressed on the cell membranes of most carcinomas, and an antibody conjugated to FITC fluorophores prepared against CD44, another cell membrane protein expressed on tumors of epithelial origin, were used. EpCAM and CD44 were diluted to a working concentration of 1:200 and introduced into the device with a flow rate of 125 μL/hr. Isotype controls for each antibody were purchased with the same fluorophore to indicate nonspecific binding in the device. Fluorescent imaging was performed by z-axis plane evaluation with 20 μm spacing showing both regional specificity of the tumor tissue and full perfusion throughout the whole FNAB tumor sample over a 24-hour period. After a 4-hour perfusion of antibody solution of both EpCAM and CD44, the antibodies could be seen throughout the FNAB tumor sample. FNAB tumor sample show slight changes both in the concentrations and where both EpCAM and CD44 are located throughout the tumor after 12 and 24-hour perfusions (S3 Fig). Results indicated that the isotype controls did not bind to the FNAB tumor fragments indicated by the absence of fluorescence throughout the FNAB tumor sample (Fig 3A). EpCAM and CD44 antibodies did bind distinctively throughout the FNAB tumor sample as seen through multiple z-axis images with 40 μm from the surface of the tumor (z/5), through the interior of the tumor (z/7 and z/9), with completion on the opposing side, 30μm from the surface (z/11) (Fig 3B). Binding was specifically mediated by recognition through protein-protein interactions as opposed to being inadvertently forced into the tissue by mechanical forces generated by flow in a nonspecific fashion.

### Assessment of tumor viability

Next, we sought to determine how long fresh FNAB tumor samples would remain viable in the device and also to characterize the working limitations of the viability dye, Calcein AM, and apoptotic antibody stain, CF594-AnnexinV. The results from the viability experiments were then used to determine the optimal length of a drug treatment trial for later experiments. FNAB tumor fragments were each placed in a channel of the device and perfused with circulating cell culture media with 5 μM Calcein AM Green and 5 μM CF594-AnnexinV, as previously described in the methods section. A baseline *Vi* analysis was performed on each FNAB tumor fragment after 12 hours of being in the device to determine the ratio of viable cells to apoptotic cells in each FNBA tumor sample. Those fragments with a *Vi* of <0.9 were deemed as nonviable and rejected from further analysis. FNAB tumor fragments were then continuously measured each day over a total period of 10 days. It was found, with all samples, that after 6 days in device the *Vi* decreased steadily with a sharp decrease in the *tRFUs* for the Calcein AM and a small less dramatic fluxuation in the *tRFUs* for the CF594-AnnexinV. Calcein AM is a cell-permeant dye that can be used to determine cell viability in eukaryotic cells. Modification of carboxylic acids with AM ester groups results in an uncharged molecule that can permeate cell membranes. Once inside the cell, the lipophilic blocking groups are cleaved through hydrolysis by nonspecific esterases in the cytoplasm of metabolically active and healthy cells, resulting in a charged form that fluoresces. CF594-AnnexinV is a 35–36 kDa protein that has a high affinity for phosphatidylserine. In normal membrane intact viable cells, phosphatidylserine is located on the cytoplasmic surface of the cell membrane. In apoptotic cells, phosphatidylserine is translocated from the inner to the outer leaflet of the plasma membrane, making itself available for AnnexinV binding. To determine if the decreasing *tRFUs* for Calcein AM was due to a decline in FNAB tumor sample viability or the working limitations of the Calcein AM dye, we re-challenged the system in several ways. First, we added fresh FNAB tumor fragments to the channels containing the Calcein AM Green and CF594-AnnexinV solution. The newly added fresh FNAB tumor fragments did not take up the Calcein AM Green dye. Next, we re-challenged the FNAB tumor fragments with CalceinAM Blue by adding a 5 μM concentration of Calcein AM Blue to the original circulating 5 μM Calcein AM Green solution. Calcein AM Blue was added to rule out if the fluorescent capability of Calcein AM Green had extinguished either by photo-bleaching due to continuous monitoring over the course of experiment and or quenching by use of the CF594-AnnexinV fluorophore. The FNAB tumor fragments did not take up the Calcein AM Blue. We then added a second round of fresh FNAB tumor fragments to the same channels. The second round of newly added fresh FNAB tumor fragments, without prior exposure to the original Calcein AM Green dye, showed a dramatic increase of Calcein AM Blue uptake into cells resulting in an increase of intensity values after a 4-hour period of incubation. Last, to test substrate concentration limitations of CalceinAM at a 5 μM concentration over the 10-day experiment, we flushed the system from the re-circulating Calcein AM and CF594-AnnexinV dyes and reintroduced newly made 5 μM CalceinAM Green and 5 μM CF594-AnnexinV to the existing, previously exposed, FNAB tumor fragments in channels and also added fresh FNAB fragments to the channel. The intensity values for Calcein AM Green increased slightly for the previously exposed FNAB fragments but only after a 48-hour period of exposure and in a limited number of cells in isolated regions of the FNAB tumor fragments. The newly added FNAB fragments resulted in the uptake of dyes with strong intensity. Experiments and results are summarized in Table 1. Results indicated that the fluorophore, in the original solution containing Calcein AM Green, had extinguished and was not able to be detected. Also, previously administered Calcein AM competitively blocked its cellular targets from binding newly added Calcein AM Green or Calcein AM Blue demonstrating that a working concentration of 5 μM was adequate for continuous binding in viable cells. In addition, it was also seen that FNAB tumor samples sustained in the system over a 10-day period, continued to take up newly added Calcein AM dye when new cells were populated in the FNAB tumor fragments. CF594-AnnexinV continued to fluoresce in the system with increased intensity peaking after 10 days. We determined that the longevity of the fluorophore conjugated to the Calcein AM had a working imitation of 5 days either due to photo-bleaching and or quenching within the system. These results show that drug experiments should ideally be performed within 5 days of placing the tumor tissue in the microfluidics device.

**Table 1:** Calcein AM dye validation and FNAB sample viability assessment.

	Original FNAB	Newly Added FNAB (without prior exposure)
**Original Calcein AM Green/AnnexinV Solution**	Decreased green intensity after 5 days	No dye uptake
**Original Calcein AM Green/AnnexinV Solution + CalceinAm Blue Solution**	No blue dye uptake	Increased blue intensity
**Flushed + New Calcein AM Green/AnnexinV Solution**	Minimal green intensity	Increased green intensity

### Drug treatment with staurosporine

**Fig 4:**
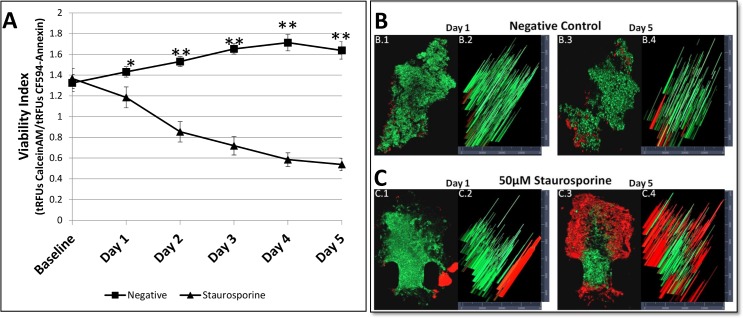
Comparison of the calculated viability index of the tumor FNAB samples during 5 days of treatment. (A) Comparison of the mean averaged VI of negative control group and staurosporine treated group from the baseline over the 5 day exposure period with n = 18 within each group. The difference in means between each treatment group was statistically significant, except for the baseline. (B.1, B.3) A 3-dimensional view of a representative FNAB sample from the negative control group at day 1 and day 5 respectively. (B.2, B.4) Histogram of the relative intensity values across the negative control FNAB samples for day 1 and day 5, respectively. (C.1, C.3) A 3-dimensional view of a representative FNAB sample from the 50 μM staurosporine treated group at day 1 and day 5 respectively. (C.2, C.4) Histogram of the relative intensity values across the 50 μM staurosporine treated FNAB samples for day 1 and day 5 respectively.

Patient derived lung adenocarcinoma FNAB tumor samples taken from xenograft mice were injected into each channel of a device and perfused with RPMI media with Calcein AM viability dye and apoptotic CF594-AnnexinV antibody dye, as previously described, and evaluated for baseline health over the course of a 12-hour period to assess for viability and apoptosis prior to start of each experiment. After the 12-hour viability assessment, with samples showing a *Vi* ratio >0.9, with approximately as many viable cells in the FNAB tumor sample than apoptotic cells, FNAB tumor fragments were then exposed to their assigned experimental condition. The test groups included a negative control group, conditioned with media and 0.1% DMSO and a drug treatment group, conditioned with 50 μM staurosporine. Each experiment was run continuously for a treatment period of 5 days. Each condition was run in triplicate for every device session tested totaling 6 experimental sessions (n = 18; each treatment group). Images were taken of each FNAB tumor fragment for every 24-hour time point over the course of 5 days. The images were later analyzed for the *tRFUs* of Calcein AM and CF594-AnnexinV. A viability index (*Vi*) was calculated for each sample at every time point and then averaged across all samples within a group for each time point. Each group was evaluated by a repeated measures ANOVA analysis for Within-Subjects Effects calculated across the 5-day testing period. A Greenhouse-Geisser correction was performed for violation against sphericity. Results for each group indicated a significant linear change over the course of the 5-day experiment (p = 0.003) with increased standard error at days 4 and 5 for the negative control group and days 1, 2 and 3 for the staurosporine treatment group. Increased standard error during those days was due to greater variability in *Vi* across samples within a treatment group. The means of each group were then compared at each time point for statistical significance. On average, the *Vi* for the negative control groups increased as the testing session continued over the course of 5 days, but decreased for the staurosporine treatment group (Fig 4A) with a statistical significance of p = 0.0355 for day 1 and p = 0.0001 for days 2–5. Fig 4B shows a three-dimensional view of the representative FNAB tumor fragments from both the negative control groups (Fig 4B.1 and Fig 4B.3) and the staurosporine treated groups (Fig 4C.1 and Fig 4C.3) at days 1 and day 5. Each view shows the ratio of viable cells (green) to apoptotic cells (red) and also how they are spatially organized and distributed within the FNAB tumor fragments. The representative negative control fragment at day 1 (Fig 4B.1) has a greater concentration of viable cells throughout the FNAB tumor fragment than the concentration of CF594-AnnexinV antibody protein on apoptotic cells, found mostly at the periphery of the sample. At day 5, (Fig 4B.3) the negative control FNAB tumor fragment has an increase in apoptotic cells at the periphery but with a majority of the cells within the FNAB tumor fragment being viable. The staurosporine treated sample on day 1 (Fig 4C.1) is highly populated with green viable cells with a distinct subpopulation of apoptotic cells in the right periphery of the sample. At day 5 (Fig 4C.3), the apoptotic subpopulation of cells seen in day 1 was lost and the majority of the viable cells seen in day 1, overtime, became apoptotic in day 5. Apoptotic cells at day 5 were located both in the periphery and the center of the FNAB tumor fragment. Fig 4B.2, 4B.4, 4C.2 and 4C.4, show a histogram of the relative intensity values across both the x-axis and y-axis for each of the representative FNAB tumor fragments. Each spike in color is measured across the corresponding axis for an intensity value. On day 1, the negative control (Fig 4B.2) had an average of 2500–3000 relative fluorescent units (RFUs) of green fluorescent protein (GFP) representing viability, Calcein AM staining, and 500–1000 RFUs of Texas Red (TR) representing apoptosis CF594-AnnexinV staining On day 5, the negative control group (Fig 4B.4) still had an average 2500–3000 RFUs of GFP with an average of 1000–2000 RFUs of TR. The percent of apoptotic cells at day 1 for the negative control group was <5% of the total volume of the tumor compared to day 5 with 20% apoptotic cells. On day 1, the Staurosporine treated group (Fig 4C.2) had an average 2000–2500 RFUs of GFP with an average of 500–1000 RFUs of TR. On day 5, the Staurosporine treated group (Fig 4B.4) had an average 1000–2000 RFUs of GFP with an average of 2500–3000 RFUs of TR. The percent of apoptotic cells at day 1 for the Staurosporine treated group was <5% of the total volume of the tumor compared to day 5 with >70% of apoptotic cells.

## Discussion

The aim of this work was to demonstrate that an engineered microfluidic device with the ability to trap FNAB tumor samples within a specifically spaced configuration of posts had the potential to maintain the viability of the tumor sample while supporting the integrity of the tumor microenvironment and thus support a more relevant and accurate assessment of drug response. The long-term goal is to use the device for personalized cancer therapy to determine the most effective drug and drug combinations for each patient. First, we validated the multi-physics model of fluid flow by quantitatively measuring the velocity within the channel of the device (S1 Text). Next, we used this data to extrapolate the shear stress on the FNAB tumor fragments in trap and concluded that with a flow rate of 125 μL/hr, tumor fragments that occluded less than 70% of the channel width experienced an average shear stress less than 0.4 dynes/cm^2^; a shear stress below the physiological critical threshold of 0.4 dynes/cm2 [38, 50], with the largest shear stress located on the posts (S1 Text). Next, we determined that the FNAB tumor fragments in trap experience full interstitial flow and therefore full drug perfusion demonstrated by the even distribution and uptake the drug, doxorubicin HCl, throughout the entire FNAB tumor fragment in channel. Additional findings further demonstrated even drug exposure upon delivery throughout the FNAB tumor fragments indicated by similar temporal activities such as nuclear fragmentation and the production of apoptotic bodies, indicative of apoptosis occurring throughout all areas of the sample. When we tested a larger molecule antibody for full sample perfusion, results were similar in showing penetration of the antibodies throughout the FNAB tumor fragments without nonspecific binding seen throughout the z-axis images. These findings are important for the indication of the microfluidic system having the diverse capability to evaluate for large molecule antibody based targeted therapies. Herein, we demonstrated a cytotoxic drug model using a known protein kinase C inhibitor, staurosporine, [41] to optimize the microfluidic device towards a potential clinically relevant chemosensitivity assay. All FNAB tumor samples were assessed for their viability at the onset of each experiment before randomly assigned to a treatment group. With the onset of the 5-day experiment, samples with a baseline *Vi* < 0.9 were deemed necrotic or nonviable and were rejected from the experiment. For days 1 through 5, there was a significant difference in the viability over death between the non-treated and treated groups. Although, each FNAB tumor fragment showed slight variability within groups in how they responded to treatment over the course of 5 days, there was a significant difference with the change of viability and death over the course of 5 days for each FNAB tumor sample between the non-treated and treated groups. With the ability to evaluate viability and death in real-time, our platform affords the user to assess FNAB tumor samples at the onset of treatment and to evaluate its response to drug treatment overtime. Real-time monitoring of treatment is a powerful tool especially for drugs that may be cytostatic or when in combinatorial treatment with cell cycle inhibitors. Further, the platform presents the ability for users to assess different areas of the FNAB tumor sample and with coordinating protein markers such as EpCAM and CD44 to know which cells are undergoing cell death or continued viability and evasion of drug response.

The clinical process of deciding a chemotherapy regime is based on empirical information from clinical trials from patients which ignores biological individuality of the tumor [51]. Patient-to-patient response is variable with anti-cancer drugs showing a high degree of variation due to each individual patient's tumor being genotypically and phenotypically different [52]. For treatment regimens to be more effective and personalized to each patient, chemosensitivity assays need to be tailored to evaluate the patient's cancer and how it responds to chemotherapy. Personalized information can better assist doctors to select effective chemotherapy regimens for individual patients. Various chemosensitivity assays used in clinic have been comprehensively reviewed [11, 12] but have been developed to assay chemosensitivity on an isolated subset of cells expanded from a tumor biopsy and cultured as a monolayer on a well plate. To accurately assess the efficacy of chemotherapy among patients, a physiologically meaningful model should be used that incorporates not only the cancer cells but also its microenvironment with its supporting stromal components. The platform must be stable with the culture environment well-defined.

Current chemosensitivity assays utilize cells cultured as a monolayer in two-dimensional models that lack the true representation of the three-dimensional microenvironment and cell to cell interactions seen in vivo. Cancer cells grown in three-dimensional environments often show more resistance to cytotoxic agents than those grown in two-dimensional cultures [8]. Formation of polarized, three-dimensional structures confers protection to cell death where in contrast, nonpolarized structures are sensitive to induction of apoptosis. Weaver et al. found that epithelial tumors that express 4-integrins have the potential to acquire resistance to exogenous death stimuli if they are given the appropriate spatial and biochemical cues from the microenvironment. Therefore, regardless of the genetic makeup, the rate of growth, or the malignant status, resistance correlates with ECM composition, hemidesmosome-dependent polarity, and nuclear factor kappa B activation [6].

A further drawback with in vitro chemosensitivity assays are the poorly defined culture conditions caused by chemical gradients in conventional static cell culture formats such as the well plate. In static well plate cell culture, support media is manually introduced or replaced periodically in a batch-wise manner. Such a nonhomogeneous and fluctuating culture environment may complicate the investigation and quantification of a cellular response to drug conditions as cellular physiology is fairly sensitive to the extracellular conditions [22]. A cell culture system using continuous fluid perfusion provides circulation of nutrients and waste maintaining a stable homogeneous culturing environment [13, 53]. Secondly, three-dimensional models do not have a homogeneous microenvironment and therefore, culturing cells in three dimensions crucially perturbs the way the cells respond to chemotherapeutic agents [54]. In cases where the three-dimensional tissue’s radius exceeds 400 μm, concentration gradients are evident where the available oxygen, metabolites, or therapeutic agents form inward gradients while that of carbon dioxide or excreted cell waste, or the acidity of extracellular fluid form outward gradients [55–57]. These gradients, and the downstream consequences that follow them, form the basis for the increased drug resistance that may be observed specifically in three-dimensional system[58]. Importantly, such gradients do exist within in vivo tumor tissues, and they interfere with the efficacy of a chemotherapeutic treatment [58]. Additionally, cells in a two-dimensional model often experience shear stress that are non-physiological and are harmful for cell viability. Chang et al. found that incubation under static conditions for 24 or 48 hours led to G0/G1 arrest; in contrast, shear stress (12 dynes/cm2) induced G2/M arrest [37]. Specifically, induced shear stress increased expressions of cyclin B1 and p21CIP1 and decreased expressions of cyclins A, D1, and E, cyclin-dependent protein kinases (Cdk)-1, -2, -4, and -6, and p27KIP1 as well as a decrease in Cdk1 activity. Also, shear-induced sensitization directly correlates with the application of fluid shear stress, and TRAIL-induced apoptosis increases in a fluid shear stress force- and time-dependent manner that is caspase dependent [50]. Therefore, to accurately assess the efficacy of chemotherapy among patients, chemosensitivity assays need to be a physiologically meaningful model that are stable and well-defined incorporating a three-dimensional microenvironment with its ability to foster cell-to-cell interactions that play important roles to determine its cellular fate as they undergo proliferation, differentiation or apoptosis.

The concept of microfluidics offers a low volume, miniaturized method for culturing and studying cellular and tissue concepts in a stable, well-defined, and more biologically relevant culture environment. Conversely, more traditional methods of cell culturing such as well plate assays are relatively large in scale requiring several hundreds of microliters to milliliters in volume with a greater demand of reagents and requiring a larger sampling population cells and or tissue. With the progress in microfabrication and microfluidic technology, microfluidic systems have been progressively used as versatile cell culture tools for various research purposes. The application of microfluidic cell culture has particularly gained more interest for drug research. Current studies of cancer dynamics within microfluidic systems have principally focused on evaluating the steps involved in cancer cell metastasis, with a benefit of these studies being that these factors make ideal biomarkers in many instances. In such studies, tumor cells have been exposed to differing shear stresses and chemokine gradients, parameters that change in capillaries in vivo, to study factors thought to be involved in generating circulating tumor cells [59, 60]. Others have examined the site of attachment and adhesion itself through the use of isotropic silicon microstructures and matrigel lined with human microvascular endothelial cells—highlighting the biomechanical properties of normal and cancer cells [61, 62]. A third group of studies have examined cell deformability, to distinguish between normal, benign, and metastatic cancer cells using antibody-coated microchannels, to develop a test for metastatic disease [63, 64]. Several of these research groups developed these microfluidic devices with chambers, posts, microbubble array, or microwells to analyze single cell behavior or trap cells in cluster, which would eventually grow into spheroids. Single cell two-dimensional models have their limitations and spheroids, although three-dimensional in conformation, rely on co-culturing cell lines that do not represent a model of personalized treatment for patients. Hattersley et al. successfully demonstrated the ability to model cell death by chemotherapeutic agents in head and neck squamous cell carcinoma biopsies although analysis was performed offline without real-time microscopic evaluation of the tumor microenvironment with dependency of dissemination and lysis of the biopsy sample [35].

Herein, we described a microfluidic platform with the ability to sustain FNAB tumor samples with full interstitial perfusion for the real-time monitoring of chemotherapy. The trapping posts within the device were strategically engineered to be spaced to retain the FNAB in channel during flow and to allow interstitial flow within the channel fostering full fluid perfusion of the FNAB tumor fragments; if spaced too wide the FNAB tumor sample would be lost in flow, if spaced too close back pressure would be created with most of the fluid traveling around the outside of the FNAB rather than interstitially through the FNAB tumor sample. Results from both the fluorescent bead assay in flow and the doxorubicin HCl drug perfusion study indicated that the FNAB tumor fragments are in close contact with the fluid front on all sides of the sample. The doxorubicin HCl drug perfusion experiment exemplifies advective properties with drug uptake throughout the whole sample in a simultaneous manner rather than as a progressive gradient in a diffusion manner with subsequent apoptotic events happening simultaneously throughout the entire sample. Additionally, the large molecule antibody experiment demonstrated antibodies bound to conjugate membrane proteins throughout the interior of the FNAB tumor samples shown through various z-axis images. These findings validate and further exemplify the advective properties of bulk fluid flow within the channel rather than dependence on diffusion alone. Traps engineered in other microfluidic devices have similar orientations but different dimensions and spacing that do not offer full interstitial flow and therefore resulting in chemical gradients therefore experiencing unstable nonhomogeneous microenvironments. Khoury et al. found that embryonic stem cell aggregates can asymmetrically differentiate in relation to their location within the microfluidic trap, and in close correlation with the state of the culture medium potentiating nutrient concentration gradients. Cells that were shielded from flow were exposed to lower nutrient levels and were most likely exposed to higher concentrations of excreted factors. Reduced nutrition and serum starvation has been shown to reduce proliferation rates and hinder cell cycle progression [65].

While two-dimensional cell culture models have their limitations, in vivo mouse models suffer the disadvantages of expense and the limitations of multiplexing for statistical relevance with unfavorable ethical concerns. Microfluidic devices have been widely used for investigation of cellular responses and viability. Microfluidic devices offer controlled environments for regulatory signals such as cytokines and physical factors like hydrodynamic shear with low burden on reagent use. Unlike a static cell culture, a perfusion cell culture model can provide a system for continuous nutrient supply and waste removal, thereby maintaining a more stable culture environment [14, 22]. We have created a stable and quantifiable fluid delivery system that provides circulation found in the body with the relevant pressures and shear experienced by solid tumors in situ.

## Conclusion

Herein we demonstrated a microfluidic device capable of trapping and maintaining the viability of FNAB tumor samples for the investigation of chemotherapy efficacy. We have shown that the fluid dynamics of the device operate at values that are physiologically relevant without insult or harm to the sample. Also, we demonstrated that the FNAB samples are fully exposed to drug infusion without the production of gradients so that a full and robust determination of drug effect may be ascertained from the viability results. Microfluidics can be a valuable tool for cancer investigation and ultimately clinicians in providing insight into the best treatment options tailored to each patient. The device has the potential to predict patient response before initiation of therapy that would help to design the most efficient therapeutic regimen for individual patients while sparing others who are not likely to benefit from the given therapy.

## Supporting Information

S1 Text Simulation of fluid flow in system using COMSOL multi-physics software. (DOCX)

S1 Fig Simulation of shear stress on a tumor trapped inside a channel by the posts. (A) Surface plot of the shear rate of a tumor occluding 50% of the channel at a flow rate of 125 μL/hr with an average shear stress of 0.257 dynes/cm^2^ around the tumor boundary. (B) Average shear stress experienced by the tumor at varying flow rates using a 50% occlusion model of tumor in channel. (C) Average shear stress experienced by tumor with occlusions of tumor in channel ranging from 50%–90% at a flow rate of 125 μL/hr.
(TIF)

S2 Fig COMSOL multi-physics software simulation of fluid velocity in channel through the device trap. (A) The simulation for velocity showing laminar fluid flow at a flow rate of 125 μL/hr. (B) 10x magnification of a Texas Red fluorescent image superimposed on a phase contrast image of 1 μm diameter fluorescing beads flowing through channel at a flow rate of 125 μL/hr. Image shows laminar flow around the trapping posts in channel. (C) 10x magnification of a Texas Red fluorescent image superimposed on a phase contrast image of fluorescing beads flowing at a rate of 125 μL/hr round a FNAB tissue sample of lung adenocarcinoma. (D) 10x magnification of a Texas Red fluorescent image superimposed on a phase contrast image of fluorescing beads flowing at a rate of 125 μL/hr round a FNAB tissue sample of melanoma. (E) 10x magnification of a Texas Red fluorescent image superimposed on a phase contrast image of fluorescing beads flowing at a rate of 125 μL/hr around a FNAB tissue sample of bladder squamous cell carcinoma.
(TIF)

S3 Fig Twenty-four hour time point evaluation of antibody perfusion through the tumor FNAB on device. (A) 10x phase contrast image of FNAB sample in trap of device and 10x fluorescent z-axis images (z5, z7, z9, z11) 2 hours post the staining procedure using EpCAM (red-Cy5) and CD44 (green-FITC). (B) 10x phase contrast image of FNAB sample in trap of device and 10x fluorescent z-axis images (z5, z7, z9, z11) 4 hours post the staining procedure using EpCAM (red-Cy5) and CD44 (green-FITC). (C) 10x phase contrast image of FNAB sample in trap of device and 10x fluorescent z-axis images (z5, z7, z9, z11) 12 hours post the staining procedure using EpCAM (red-Cy5) and CD44 (green-FITC). (D) 10x phase contrast image of FNAB sample in trap of device and 10x fluorescent z-axis images (z5, z7, z9, z11) 24 hours post the staining procedure using EpCAM (red-Cy5) and CD44 (green-FITC).
(TIF)

## References

[1] Society AC. Cancer Facts and Figures 2014 2014 [cited 2014 12/13/2014]. Available from: http://www.cancer.org/research/cancerfactsstatistics/cancerfactsfigures2014/index.

[2] Costa FF, Seftor EA, Bischof JM, Kirschmann DA, Strizzi L, Arndt K, et al Epigenetically reprogramming metastatic tumor cells with an embryonic microenvironment. Epigenomics. 2009;1(2):387–98. 10.2217/epi.09.25 20495621 PMC2872497

[3] Sharma SV, Lee DY, Li B, Quinlan MP, Takahashi F, Maheswaran S, et al A Chromatin-Mediated Reversible Drug-Tolerant State in Cancer Cell Subpopulations. Cell. 2010;141(1):69–80. 10.1016/j.cell.2010.02.027 20371346 PMC2851638

[4] Garnett MJ, Edelman EJ, Heidorn SJ, Greenman CD, Dastur A, Lau KW, et al Systematic identification of genomic markers of drug sensitivity in cancer cells. Nature. 2012;483(7391):570–5. 10.1038/nature11005 22460902 PMC3349233

[5] Straussman R, Morikawa T, Shee K, Barzily-Rokni M, Qian ZR, Du J, et al Tumour micro-environment elicits innate resistance to RAF inhibitors through HGF secretion. Nature. 2012;487(7408):500–4. 10.1038/nature11183 22763439 PMC3711467

[6] Weaver VM, Lelièvre S, Lakins JN, Chrenek MA, Jones JCR, Giancotti F, et al β4 integrin-dependent formation of polarized three-dimensional architecture confers resistance to apoptosis in normal and malignant mammary epithelium. Cancer cell. 2002;2(3):205–16. 12242153 10.1016/s1535-6108(02)00125-3 PMC2788997

[7] Yamada KM, Cukierman E. Modeling Tissue Morphogenesis and Cancer in 3D. Cell. 2007;130(4):601–10. 10.1016/j.cell.2007.08.006 17719539

[8] Torisawa Y-s, Shiku H, Yasukawa T, Nishizawa M, Matsue T. Multi-channel 3-D cell culture device integrated on a silicon chip for anticancer drug sensitivity test. Biomaterials. 2005;26(14):2165–72. 10.1016/j.biomaterials.2004.05.028 15576192

[9] Huang S-B, Wang S-S, Hsieh C-H, Lin YC, Lai C-S, Wu M-H. An integrated microfluidic cell culture system for high-throughput perfusion three-dimensional cell culture-based assays: effect of cell culture model on the results of chemosensitivity assays. Lab on a Chip. 2013;13(6):1133–43. 10.1039/c2lc41264k 23353927

[10] Barry MA, Behnke CA, Eastman A. Activation of programmed cell death (apoptosis) by cisplatin, other anticancer drugs, toxins and hyperthermia. Biochemical Pharmacology. 1990;40(10):2353–62. 2244936 10.1016/0006-2952(90)90733-2

[11] Herzog TJ, Krivak TC, Fader AN, Coleman RL. Chemosensitivity testing with ChemoFx and overall survival in primary ovarian cancer. American Journal of Obstetrics and Gynecology. 2010;203(1):68.e1–.e6. 20227055 10.1016/j.ajog.2010.01.059

[12] Bosserman L, Prendergast F, Herbst R, Fleisher M, Salom E, Strickland S, et al The Microculture-Kinetic (MiCK) Assay: The Role of a Drug-Induced Apoptosis Assay in Drug Development and Clinical Care. Cancer Research. 2012;72(16):3901–5. 10.1158/0008-5472.CAN-12-0681 22865459

[13] Wu M-H, Chang Y-H, Liu Y-T, Chen Y-M, Wang S-S, Wang H-Y, et al Development of high throughput microfluidic cell culture chip for perfusion 3-dimensional cell culture-based chemosensitivity assay. Sensors and Actuators B: Chemical. 2011;155(1):397–407.

[14] Wu M, Urban JG, Cui Z, Cui Z. Development of PDMS microbioreactor with well-defined and homogenous culture environment for chondrocyte 3-D culture. Biomedical Microdevices. 2006;8(4):331–40. 10.1007/s10544-006-9597-y 16917663

[15] Maitland ML, DiRienzo A, Ratain MJ. Interpreting Disparate Responses to Cancer Therapy: The Role of Human Population Genetics. Journal of Clinical Oncology. 2006;24(14):2151–7. 10.1200/JCO.2005.05.2282 16682733

[16] Müerköster S, Wegehenkel K, Arlt A, Witt M, Sipos B, Kruse M-L, et al Tumor Stroma Interactions Induce Chemoresistance in Pancreatic Ductal Carcinoma Cells Involving Increased Secretion and Paracrine Effects of Nitric Oxide and Interleukin-1β. Cancer Research. 2004;64(4):1331–7. 14973050 10.1158/0008-5472.can-03-1860

[17] Tárnok A, Bocsi J, Brockhoff G. Cytomics–importance of multimodal analysis of cell function and proliferation in oncology. Cell Proliferation. 2006;39(6):495–505. 10.1111/j.1365-2184.2006.00407.x 17109634 PMC6496464

[18] Wlodkowic D, Skommer J, Darzynkiewicz Z. Cytometry in cell necrobiology revisited. Recent advances and new vistas. Cytometry Part A. 2010;77A(7):591–606. 10.1002/cyto.a.20889 PMC2975392 20235235

[19] El-Ali J, Sorger PK, Jensen KF. Cells on chips. Nature. 2006;442(7101):403–11. 10.1038/nature05063 16871208

[20] Feng X, Du W, Luo Q, Liu B-F. Microfluidic chip: Next-generation platform for systems biology. Analytica Chimica Acta. 2009;650(1):83–97. 10.1016/j.aca.2009.04.051 19720178

[21] Whitesides GM. The origins and the future of microfluidics. Nature. 2006;442(7101):368–73. 10.1038/nature05058 16871203

[22] Wu M-H, Urban JPG, Cui ZF, Cui Z, Xu X. Effect of Extracellular pH on Matrix Synthesis by Chondrocytes in 3D Agarose Gel. Biotechnology Progress. 2007;23(2):430–4. 10.1021/bp060024v 17286385

[23] Kim L, Toh Y-C, Voldman J, Yu H. A practical guide to microfluidic perfusion culture of adherent mammalian cells. Lab on a Chip. 2007;7(6):681–94. 10.1039/b704602b 17538709

[24] Ling Y, Rubin J, Deng Y, Huang C, Demirci U, Karp JM, et al A cell-laden microfluidic hydrogel. Lab on a Chip. 2007;7(6):756–62. 10.1039/b615486g 17538718

[25] Nevill JT, Cooper R, Dueck M, Breslauer DN, Lee LP. Integrated microfluidic cell culture and lysis on a chip. Lab on a Chip. 2007;7(12):1689–95. 10.1039/b711874k 18030388

[26] Sung KE, Yang N, Pehlke C, Keely PJ, Eliceiri KW, Friedl A, et al Transition to invasion in breast cancer: a microfluidic in vitro model enables examination of spatial and temporal effects. Integrative Biology. 2011;3(4):439–50. 10.1039/c0ib00063a 21135965 PMC3094750

[27] Abbott A. Cell culture: Biology's new dimension. Nature. 2003;424(6951):870–2. 10.1038/424870a 12931155

[28] Griffith LG, Swartz MA. Capturing complex 3D tissue physiology in vitro. Nat Rev Mol Cell Biol. 2006;7(3):211–24. 10.1038/nrm1858 16496023

[29] Ng CP, Pun SH. A perfusable 3D cell–matrix tissue culture chamber for in situ evaluation of nanoparticle vehicle penetration and transport. Biotechnology and bioengineering. 2008;99(6):1490–501. 10.1002/bit.21698 17969174 PMC2796126

[30] Bissell MJ, Rizki A, Mian IS. Tissue architecture: the ultimate regulator of breast epithelial function. Current Opinion in Cell Biology. 2003;15(6):753–62. 14644202 10.1016/j.ceb.2003.10.016 PMC2933200

[31] Lukashev ME, Werb Z. ECM signalling: orchestrating cell behaviour and misbehaviour. Trends in Cell Biology. 1998;8(11):437–41. 9854310 10.1016/s0962-8924(98)01362-2

[32] Even-Ram S, Yamada KM. Cell migration in 3D matrix. Current Opinion in Cell Biology. 2005;17(5):524–32. 10.1016/j.ceb.2005.08.015 16112853

[33] Golden AP, Tien J. Fabrication of microfluidic hydrogels using molded gelatin as a sacrificial element. Lab Chip. 2007;7(6):720–5. 10.1039/b618409j 17538713

[34] Zhang S. Fabrication of novel biomaterials through molecular self-assembly. Nat Biotech. 2003;21(10):1171–8. 10.1038/nbt874 14520402

[35] Hattersley SM, Sylvester DC, Dyer CE, Stafford ND, Haswell SJ, Greenman J. A microfluidic system for testing the responses of head and neck squamous cell carcinoma tissue biopsies to treatment with chemotherapy drugs. Annals of biomedical engineering. 2012;40(6):1277–88. 10.1007/s10439-011-0428-9 21997391

[36] Komen J, Wolbers F, Franke HR, Andersson H, Vermes I, van den Berg A. Viability analysis and apoptosis induction of breast cancer cells in a microfluidic device: effect of cytostatic drugs. Biomedical Microdevices. 2008;10(5):727–37. 10.1007/s10544-008-9184-5 18523888 PMC2494574

[37] Chang S-F, Chang CA, Lee D-Y, Lee P-L, Yeh Y-M, Yeh C-R, et al Tumor cell cycle arrest induced by shear stress: Roles of integrins and Smad. Proceedings of the National Academy of Sciences. 2008;105(10):3927–32. 10.1073/pnas.0712353105 PMC2268796 18310319

[38] Kawai Y, Kaidoh M, Yokoyama Y, Ohhashi T. Pivotal roles of shear stress in the microenvironmental changes that occur within sentinel lymph nodes. Cancer Science. 2012;103(7):1245–52. 10.1111/j.1349-7006.2012.02289.x 22463128 PMC7659381

[39] Qazi H, Shi Z-D, Tarbell JM. Fluid Shear Stress Regulates the Invasive Potential of Glioma Cells via Modulation of Migratory Activity and Matrix Metalloproteinase Expression. PLoS ONE. 2011;6(5):e20348 10.1371/journal.pone.0020348 21637818 PMC3102715

[40] Altiok S, Mezzadra H., Jagannath S., Tsottles N., Rudek M., Abdallah N., Berman D., Forastiere A., & Gibson M. A novel pharmacodynamic approach to assess and predict tumor response to the epidermal growth factor receptor inhibitor gefitinib in patients with esophageal cancer. International Journal of Oncology. 2010;36(1):19–27. 19956829 PMC3052699

[41] Koivunen J, Aaltonen V, Peltonen J. Protein kinase C (PKC) family in cancer progression. Cancer Letters. 2006;235(1):1–10. 10.1016/j.canlet.2005.03.033 15907369

[42] Hidalgo M, Amador ML, Jimeno A, Mezzadra H, Patel P, Chan A, et al Assessment of gefitinib-and CI-1040-mediated changes in epidermal growth factor receptor signaling in HuCCT-1 human cholangiocarcinoma by serial fine needle aspiration. Molecular cancer therapeutics. 2006;5(7):1895–903. 10.1158/1535-7163.MCT-05-0525 16891476

[43] Rubio-Viqueira B, Mezzadra H, Nielsen ME, Jimeno A, Zhang X, Iacobuzio-Donahue C, et al Optimizing the development of targeted agents in pancreatic cancer: tumor fine-needle aspiration biopsy as a platform for novel prospective ex vivo drug sensitivity assays. Molecular cancer therapeutics. 2007;6(2):515–23. 10.1158/1535-7163.MCT-06-0388 17308050

[44] Leclerc E, Sakai Y, Fujii T. Cell Culture in 3-Dimensional Microfluidic Structure of PDMS (polydimethylsiloxane). Biomedical Microdevices. 2003;(2).

[45] Telang S, Nelson KK, Siow DL, Yalcin A, Thornburg JM, Imbert-Fernandez Y, et al Cytochrome c oxidase is activated by the oncoprotein Ras and is required for A549 lung adenocarcinoma growth. Molecular Cancer. 2012. 10.1186/1476-4598-11-60 PMC3546037 22917272

[46] Abdi H. The greenhouse-geisser correction. Encyclopedia of research design SAGE Publications, Thousand Oaks, CA, USA 2010.

[47] Lankelma J, Dekker H, Fernández Luque R, Luykx S, Hoekman K, Valk Pvd, et al Doxorubicin Gradients in Human Breast Cancer. Clinical Cancer Research. 1999;5(7):1703–7. 10430072

[48] de Lange JHM, Schipper NW, Schuurhuis GJ, ten Kate TK, Van Heijningen THM, Pinedo HM, et al Quantification by laser scan microscopy of intracellular doxorubicin distribution. Cytometry. 1992;13(6):571–6. 10.1002/cyto.990130604 1451589

[49] Blankenberg FG. Imaging the Molecular Signatures of Apoptosis and Injury with Radiolabeled Annexin V. Proceedings of the American Thoracic Society. 2009;6(5):469–76. 10.1513/pats.200901-001AW 19687221 PMC2731806

[50] Mitchell MJ, King MR. Fluid Shear Stress Sensitizes Cancer Cells to Receptor-Mediated Apoptosis via Trimeric Death Receptors. New journal of physics. 2013;15:015008 10.1088/1367-2630/15/1/015008 25110459 PMC4124740

[51] Hatok J, Babusikova E, Matakova T, Mistuna D, Dobrota D, Racay P. In vitro assays for the evaluation of drug resistance in tumor cells. Clinical and experimental medicine. 2009;9(1):1–7. 10.1007/s10238-008-0011-3 18818983

[52] Qi C-J, Ning Y-L, Zhu Y-L, Min H-Y, Ye H, Qian K-Q. In vitro chemosensitivity in breast cancer using ATP-tumor chemosensitivity assay. Archives of pharmacal research. 2009;32(12):1737–42. 10.1007/s12272-009-2211-0 20162402

[53] Sittinger M, Schultz O, Keyszer G, Minuth W, Burmester G. Artificial tissues in perfusion culture. The International journal of artificial organs. 1997;20(1):57–62. 9062833

[54] Kim JB. Three-dimensional tissue culture models in cancer biology. Seminars in Cancer Biology. 2005;15(5):365–77. 10.1016/j.semcancer.2005.05.002 15975824

[55] Fischbach C, Chen R, Matsumoto T, Schmelzle T, Brugge JS, Polverini PJ, et al Engineering tumors with 3D scaffolds. Nat Meth. 2007;4(10):855–60. 10.1038/nmeth1085 17767164

[56] Håkanson M, Textor M, Charnley M. Engineered 3D environments to elucidate the effect of environmental parameters on drug response in cancer. Integrative Biology. 2011;3(1):31–8. 10.1039/c0ib00074d 21049126

[57] Helmlinger G, Yuan F, Dellian M, Jain RK. Interstitial pH and pO2 gradients in solid tumors in vivo: high-resolution measurements reveal a lack of correlation. Nature medicine. 1997;3(2):177–82. 9018236 10.1038/nm0297-177

[58] Trédan O, Galmarini CM, Patel K, Tannock IF. Drug resistance and the solid tumor microenvironment. Journal of the National Cancer Institute. 2007;99(19):1441–54. 10.1093/jnci/djm135 17895480

[59] Couzon C, Duperray A, Verdier C. Critical stresses for cancer cell detachment in microchannels. European Biophysics Journal. 2009;38(8):1035–47. 10.1007/s00249-009-0506-1 19579023

[60] Walker GM, Sai J, Richmond A, Stremler M, Chung CY, Wikswo JP. Effects of flow and diffusion on chemotaxis studies in a microfabricated gradient generator. Lab on a Chip. 2005;5(6):611–8. 10.1039/b417245k 15915253 PMC2665276

[61] Du Z, Cheng KH, Vaughn MW, Collie NL, Gollahon LS. Recognition and capture of breast cancer cells using an antibody-based platform in a microelectromechanical systems device. Biomedical Microdevices. 2007;9(1):35–42. 10.1007/s10544-006-9010-x 17103049

[62] Lindström S, Mori K, Ohashi T, Andersson‐Svahn H. A microwell array device with integrated microfluidic components for enhanced single‐cell analysis. Electrophoresis. 2009;30(24):4166–71. 10.1002/elps.200900572 19938185

[63] Chaw K, Manimaran M, Tay E, Swaminathan S. Multi-step microfluidic device for studying cancer metastasis. Lab on a Chip. 2007;7(8):1041–7. 10.1039/b707399m 17653347

[64] Hou HW, Li Q, Lee G, Kumar A, Ong C, Lim CT. Deformability study of breast cancer cells using microfluidics. Biomedical Microdevices. 2009;11(3):557–64. 10.1007/s10544-008-9262-8 19082733

[65] Khoury M, Bransky A, Korin N, Konak L, Enikolopov G, Tzchori I, et al A microfluidic traps system supporting prolonged culture of human embryonic stem cells aggregates. Biomedical Microdevices. 2010;12(6):1001–8. 10.1007/s10544-010-9454-x 20665114

