# Supplementary material for: Microfluidic Biopsy Trapping Device for the Real-Time Monitoring of Tumor Microenvironment
Source: PLoS One. 2017 Jan 13;12(1):e0169797. doi: 10.1371/journal.pone.0169797 (PMC5235371; doi:10.1371/journal.pone.0169797)
Supplement: S1 Text — (DOCX) [file pone.0169797.s001.docx]

## **Simulation of fluid flow in system using COMSOL multi-physics software**

Computational modeling (COMSOL Inc., Burlington, MA) of a straight channel with posts arranged in a semi-circular staggered array was performed in three dimensions to simulate the laminar fluid flow. The Newtonian fluid dynamic module solved the Navier-Stokes equation at steady state where Table A summarizes the equations used.. The density and viscosity values were specified for water at room temperature (25°C). The time to reach steady state for flow in the channel is almost instantaneous (~0.003 s to ~0.006 s) for flow rates between 50-500 µL/hr, confirmed by independent transient laminar flow simulations using the same geometry and flow rates experimentally studied in the device. Therefore, solving the laminar flow as a stationary phenomenon versus transient flow is a reasonable approximation. The modeling parameters are listed in Table B.

Table A. Equations Employed During Computational Model.

| **Name** | **Equation** |
| --- | --- |
| Continuity Equation |  |
| Momentum Equation |  |

u, velocity; ρ, density; P, pressure; µ, viscosity.

Table B. Modeling Parameters.

| **Parameter** | **Value** | **Description** |
| --- | --- | --- |
| ρ | 998.2 kg/m^3^ | Density of water |
| µ | 1.003e-3 Pa*s | Dynamic viscosity of water |

u, velocity; ρ, density; kg/m^3^  kilogram per meter cubed; Pa*s, pascal

second.

Channel layout and dimensions were taken from the device design. A Poiseulle boundary condition was applied at the inlet, with a zero-pressure outlet and a no-slip fluid—wall interface. These boundary conditions were imposed to solve for the laminar flow. Mesh refinements were performed to minimize the flow flux between inlet and outlet. After two successive refinements, resulting in 569,335 mesh elements, the error in flow flux ranged from 1.39% to 4.80%, increasing in error with higher flow rates. Further mesh refinements were tested on simulations using flow rates > 125 μL/hr; with only a decrease in error of 0.5% leading to a 3-fold computation time increase. An iterative geometric multigrid (GMRES) algorithm solved the equations for the steady-state condition, while a direct backward differentiation formula (BDF) algorithm was required for the transient study.

To assess the effect of shear stress and channel occlusion on the FNAB tumor sample in trap, we constructed plots of the average shear rate on the tumor perimeter for various flow rates and tumor sizes. Plots were compared at multiple flow rates of 50 µL/hr through 500 µL/hr for a single channel (600 µm by 125 µm channel cross section).

## **Calculation of the velocity of the fluid flow in device with fluorescent bead assay**

To calculate the velocity fluids and trace the fluid dynamics of the device we used fluorescently conjugated beads (Molecular Probes® FluoSpheres® beads, Life Technologies). A concentration of 1x10^6^ beads/mL was made diluted in phosphate buffer. The bead solution was perfused through the device by using a peristaltic pump (Masterflex L/S Digital Drive, 100 RPM, 115/230 VAC) purchased from Cole Parmer. Flow rates of 15, 25, 65, 125, 250 and 500 µL/hr were run and the flow was monitored and recorded by fluorescent microscopy using the Zeiss Axio ObserverZ.1/ApoTome.2 imaging system. Images were taken of the fluorescent beads under flow in the 580/605nm excitation/emission spectrum. Each image frame was evaluated measuring the length of the path the bead traveled divided by the exposure time. The resulting velocities were used to validate the COMSOL simulations to determine their accuracies for shear stress of the fluids on the tumor tissue.

## **Simulation of fluid flow in microfluidic device channel using COMSOL multi-physics software**

A model of fluid flowing in the channel of the microfluidic device was designed to understand how various parameters such as flow rate, channel dimensions and tumor size would affect the FNAB tissue sample in the designed trap.

### **Shear stress**

To assess the shear stress on a tumor, we simulated a tumor, trapped inside a channel by the posts. The tumor was modeled with no-slip boundaries, which translates to no interstitial flow within the tumor. A surface plot of the shear rate of a tumor occluding 50% of the channel at a flow rate of 125 µL/hr (S1A Fig) shows the areas with largest shear rate are located by the posts, with an average shear stress of 0.257 dynes/cm^2^ around the tumor boundary. A parametric study was performed to evaluate the average shear rate experienced by the tumor at varying flow rates using a 50% occlusion model. It was found that in order to remain below a threshold of 0.4 dynes/cm2, a physiological critical threshold for shear stress [[1](#_ENREF_1), [2](#_ENREF_2)], for a tumor that occluded the channel by 50%, a flow rate of less than ~190 µL/hr was needed (S1B Fig). Further, we simulated various tumor sizes that occluded the channel between 50% - 90% at a flow rate of 125 µL/hr. At this flow rate, it was found that tumors that occluded less than 70% of the channel width experienced an average shear stress less than 0.4 dynes/cm^2^ (S1C Fig). The effect of pressure was also calculated in the system and it was found that there was a negligible pressure drop across the tumor of 5.812 pa.

### **Model Validation**

To assess the accuracy of the simulation model performed with multiphysics software licensed from COMSOL, we tested the fluid profile through the device with 1µm diameter polystyrene beads conjugated to a fluorophore with an excitation/emission wavelength of 580/605 nm. Beads were introduced into a reservoir in phosphate buffer solution for final concentration of 1x10^6^ beads/mL and pumped through the device with a re-circulating peristaltic pump at a range of flow rates between 15-500 µL/hr. These flow rates correspond to a Reynolds number range between 0.01-0.40, using ρ as the fluid density, u as the average velocity, D_H_ as the hydraulic diameter of the channel and µ as the fluid dynamic viscosity. Given that a Reynolds number below 2000 indicates laminar flow [[3](#_ENREF_3)], we concluded that the channel had laminar flow and the design did not impose turbulence. The simulation for velocity shows a profile of laminar fluid flow and no indication of eddy currents or large areas of dead volume around the tumor traps (S2A Fig). The simulation was performed at a flow rate of 125 µL/hr. S2B Fig shows an image of fluorescent beads flowing around the trapping posts; as seen in the model, similar streamlines are represented in the experiment, confirming a laminar flow around the posts. To validate the computational model, we took videos of the fluorescent beads flowing through the channel, at a distance half way between the inlet of the channel and the trapping posts and at a z-axis location of 62.5 μm, which corresponds to the center plane of the channel height. Beads flowing in the center 25% of the channel width were measured at 81.8 s and 329.5 s, which correspond to the periods of maximum velocity in the peristaltic pump pulsation cycle. The measured distances were then divided by the exposure time and averaged to yield a maximum velocity range of 0.00097 to 0.00136 m/s. Maximum velocity was measured because it yields the maximum shear rate for a given flow rate. When comparing the experimental and simulated maximum velocities, it was found that both values were in agreement, with a -3.56% error between them, showing that the computational model is a valid approximation for the fluid flow within the trapping device.

Next, we challenged the laminar flow profile by adding FNAB tumor samples of different density, shape and size to the device channels to determine if occluding flow would cause turbulent flow. FNAB tumor samples from patient derived xenograft mice were biopsied as previously explained. We tested lung adenocarcinoma, melanoma, and bladder squamous cell carcinoma. Fluorescent polystyrene beads were flown into the channel at varying flow rates between 125 – 500 µL/hr. Fluorescent imaging was performed to capture the flow profile of fluorescently conjugated polystyrene beads in channel. It was found that properties of laminar flow in the channel were sustained with the addition of FNAB tumor fragments in channel trap. By adding FNAB tumor fragments to the channel traps and therefore creating some degree of occlusion of fluid flow within the channel, higher velocities at the sidewalls of the channel were created. This was determined by measuring larger distances traveled in each frame seen by the fluorescent stream of the beads (S2C-E Fig). However, we also found that the beads in the center of the channel at the fluid interface closest to the tumor showed little to no distance traveled within frames and therefore lower velocities. These observations prompted further testing using smaller molecular weight drugs, such as Doxorubicin, to test for areas of stagnation at the fluid interface of the tumor and to further evaluate for convective properties with full fluid perfusion throughout the FNAB tumor sample.

**References**

1. Kawai Y, Kaidoh M, Yokoyama Y, Ohhashi T. Pivotal roles of shear stress in the microenvironmental changes that occur within sentinel lymph nodes. Cancer Science. 2012;103(7):1245-52. doi: 10.1111/j.1349-7006.2012.02289.x.

2. Mitchell MJ, King MR. Fluid Shear Stress Sensitizes Cancer Cells to Receptor-Mediated Apoptosis via Trimeric Death Receptors. New journal of physics. 2013;15:015008. doi: 10.1088/1367-2630/15/1/015008. PubMed PMID: PMC4124740.

3. Rott N. Note on the History of the Reynolds Number. Annual Review of Fluid Mechanics. 1990;22(1):1-12. doi: doi:10.1146/annurev.fl.22.010190.000245.
