# Supplementary figures and images for: Microfluidic Biopsy Trapping Device for the Real-Time Monitoring of Tumor Microenvironment
Source: PLoS One. 2017 Jan 13;12(1):e0169797. doi: 10.1371/journal.pone.0169797 (PMC5235371; doi:10.1371/journal.pone.0169797)

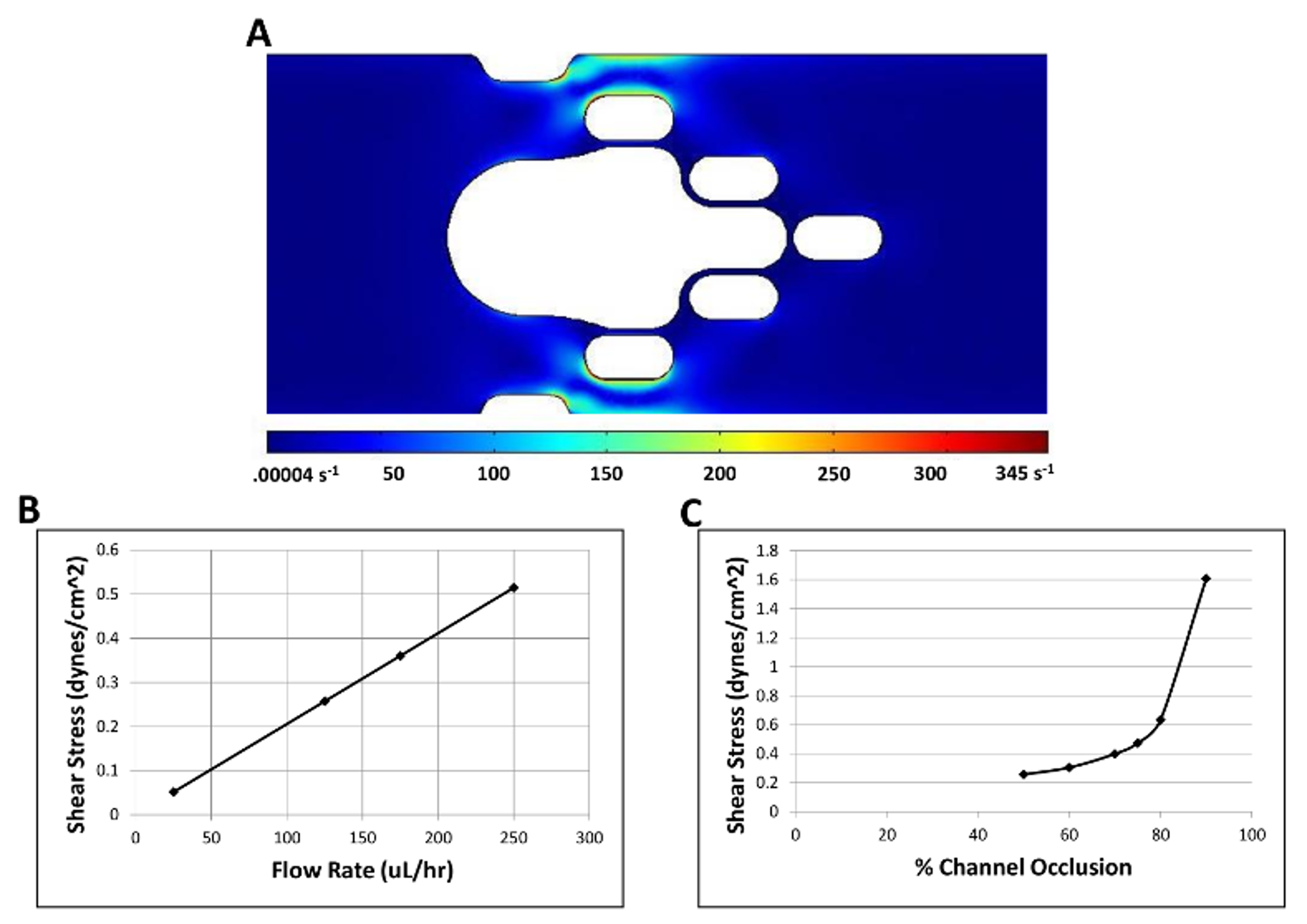

Supplement: S1 Fig — (A) Surface plot of the shear rate of a tumor occluding 50% of the channel at a flow rate of 125 μL/hr with an average shear stress of 0.257 dynes/cm2 around the tumor boundary. (B) Average shear stress experienced by the tumor at varying flow rates using a 50% occlusion model of tumor in channel. (C) Average shear stress experienced by tumor with occlusions of tumor in channel ranging from 50%–90% at a flow rate of 125 μL/hr. (TIF) [file pone.0169797.s002.tif]

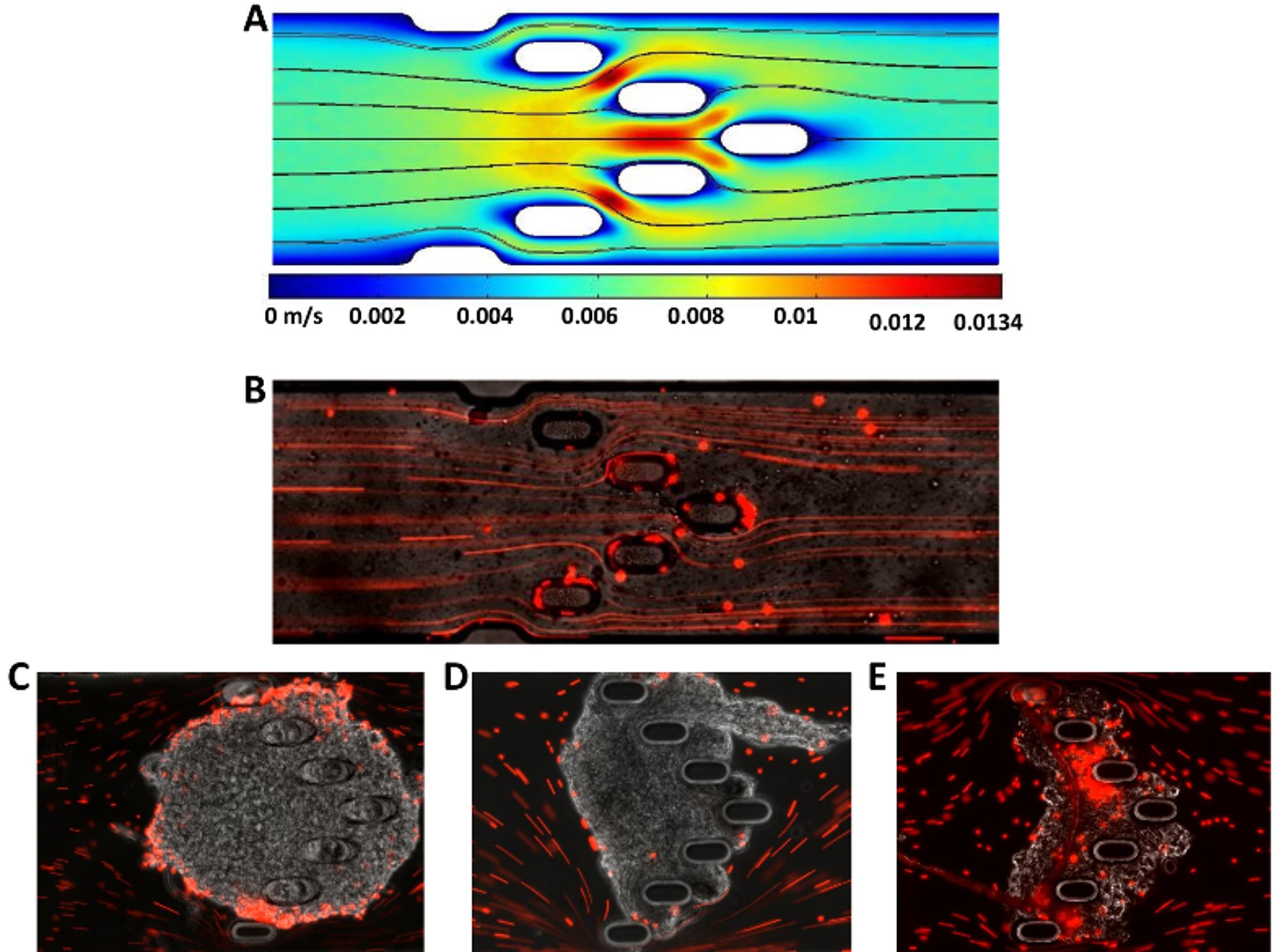

Supplement: S2 Fig — (A) The simulation for velocity showing laminar fluid flow at a flow rate of 125 μL/hr. (B) 10x magnification of a Texas Red fluorescent image superimposed on a phase contrast image of 1 μm diameter fluorescing beads flowing through channel at a flow rate of 125 μL/hr. Image shows laminar flow around the trapping posts in channel. (C) 10x magnification of a Texas Red fluorescent image superimposed on a phase contrast image of fluorescing beads flowing at a rate of 125 μL/hr round a FNAB tissue sample of lung adenocarcinoma. (D) 10x magnification of a Texas Red fluorescent image superimposed on a phase contrast image of fluorescing beads flowing at a rate of 125 μL/hr round a FNAB tissue sample of melanoma. (E) 10x magnification of a Texas Red fluorescent image superimposed on a phase contrast image of fluorescing beads flowing at a rate of 125 μL/hr around a FNAB tissue sample of bladder squamous cell carcinoma. (TIF) [file pone.0169797.s003.tif]

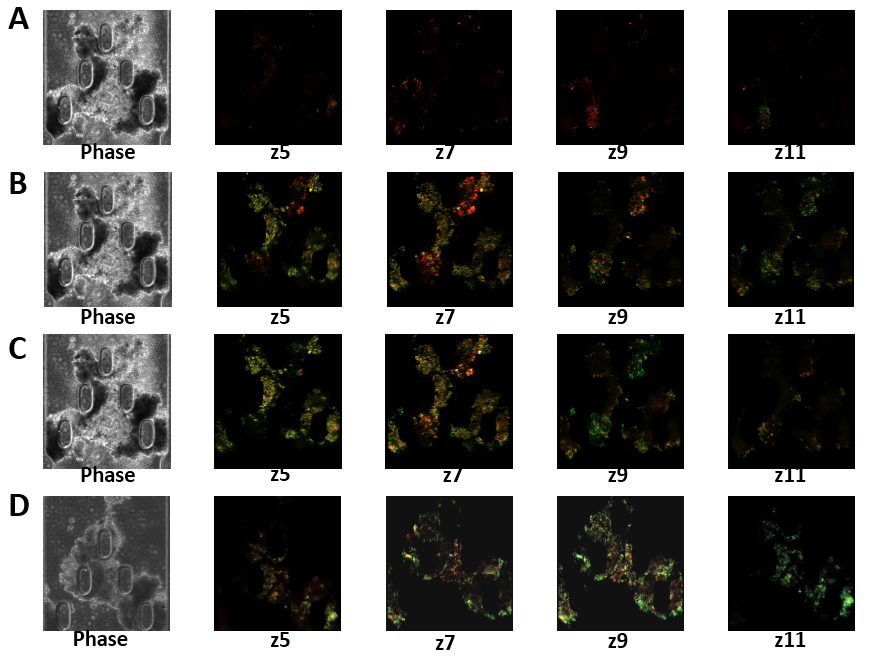

Supplement: S3 Fig — (A) 10x phase contrast image of FNAB sample in trap of device and 10x fluorescent z-axis images (z5, z7, z9, z11) 2 hours post the staining procedure using EpCAM (red-Cy5) and CD44 (green-FITC). (B) 10x phase contrast image of FNAB sample in trap of device and 10x fluorescent z-axis images (z5, z7, z9, z11) 4 hours post the staining procedure using EpCAM (red-Cy5) and CD44 (green-FITC). (C) 10x phase contrast image of FNAB sample in trap of device and 10x fluorescent z-axis images (z5, z7, z9, z11) 12 hours post the staining procedure using EpCAM (red-Cy5) and CD44 (green-FITC). (D) 10x phase contrast image of FNAB sample in trap of device and 10x fluorescent z-axis images (z5, z7, z9, z11) 24 hours post the staining procedure using EpCAM (red-Cy5) and CD44 (green-FITC). (TIF) [file pone.0169797.s004.tif]
